# *Ganoderma lucidum* polysaccharide improves rat DSS-induced colitis by altering cecal microbiota and gene expression of colonic epithelial cells

**DOI:** 10.29219/fnr.v63.1559

**Published:** 2019-02-12

**Authors:** Jinli Xie, Yanghanxiu Liu, Bohui Chen, Guangwen Zhang, Shiyi Ou, Jianming Luo, Xichun Peng

**Affiliations:** Department of Food Science and Engineering, Jinan University, Guangzhou, China

**Keywords:** *Ganoderma lucidum* polysaccharide, colitis, short-chain fatty acids, gut microbiota, colonic epithelial expression

## Abstract

**Background:**

The effects of β-glucan on colitis mice are contradictory in previous reports. As a result, it is still unclear whether there is an anti-colitis effect in *Ganoderma lucidum* polysaccharide (GLP), which is mainly composed of β-glucan. Moreover, the association between GLP function and gut microbiota remains to be elucidated.

**Objective:**

This study aimed to investigate whether GLP consumption improved rat dextran sodium sulfate (DSS)-induced colitis by regulating gut microbiota and altering colonic epithelial expression.

**Design:**

The disease activity index (DAI) scores and the cecal short chain fatty acid (SCFA) levels of DSS-induced colitis rats fed with a GLP diet (Group GLP, *n* = 6) and a control diet (Group Con, *n* = 6) were investigated and analyzed. Moreover, the profiles of gut microbiota and colonic epithelial expression were analyzed using metagenomics and transcriptomics.

**Results:**

GLP consumption significantly lowered animal DAI scores by producing more SCFAs by increasing SCFA-producing bacteria such as *Ruminococcus_1* and reducing pathogens such as *Escherichia-Shigella* in both the small intestine and cecum of rat. Moreover, GLP consumption regulated 11 genes, including six upregulated (*Ccl5*, *Cd3e*, *Cd8a*, *Il21r*, *Lck*, and *Trbv*) and five downregulated (*Ccl3*, *Gro*, *Il11*, *Mhc2*, and *Ptgs*) genes enriched in six inflammation-related Kyoto Encyclopedia of Genes and Genomes (KEGG) pathways, resulting in enhancement of immunity and reduction of inflammatory response and colonic cancer risk.

**Conclusions:**

GLP consumption alleviated DSS-induced colitis and may have potential for ulcerative colitis relief.

## Popular scientific summary

- GLP consumption in colitis rats significantly lowered the DAI and produced markedly more SCFAs in the cecum. This may be mainly due to the increase of SCFA-producing bacteria and reduction of pathogens in both small intestine and cecum.
- SCFAs and the altered gut microbiota further regulated the expression of 11 genes enriched in 6 KEGG pathways related to inflammation, resulting in the immunity enhancement, inflammatory response alleviation and colon cancer risk reduction. Therefore, GLP could alleviate DSS-induced colitis, which most closely resembles human UC and thus may have potential for UC relief.

## 

Ulcerative colitis (UC) is characterized by superficial mucosal inflammation, rectal bleeding, diarrhea, and abdominal pain (1). UC and Crohn’s disease are two forms of chronic inflammatory bowel disease (IBD), whose incidence has steadily risen all over the world, including in China, over the past 10 years (2, 3).

Intestinal microbiota have a close relationship with UC. They are thought to play a central role in the pathogenesis of IBD, as indicated by experimental mouse model studies that demonstrated that the development of IBD was due to excessive translocation of bacteria into the bowel wall or the dysregulation of bacteria in genetically susceptible hosts (4). Therefore, fecal microbiota transplantation is a promising treatment for UC, with few adverse events (5).

Furthermore, the use of prebiotics, which can enhance the survival and action of probiotic bacteria, is supported by a strong biological rationale for their therapeutic effect on IBD (6). For example, the intake of dietary fibers, the most common prebiotics, was revealed to be associated with a lower risk of UC (7). As one of the dietary fibers, *Ganoderma lucidum* polysaccharide (GLP) consists of beta-1, 3/1,6-glucan and some other carbohydrates, with beta-1,3-glucan as the main active ingredient (8). It has been reported to modulate immunity and has shown potential antitumor activity (9). Recently, our study demonstrated that GLP supplementation alleviated colorectal cancer in mice (10), but the exact effect of GLP against colitis remained to be elucidated because contradictory results of β-glucan have been reported in colitis mice (11, 12). Furthermore, no study has investigated whether the effect of GLP on colitis is associated with gut microbiota. This study aimed to investigate whether and how GLP consumption improves dextran sodium sulfate (DSS)-induced colitis in rats by regulating gut microbiota and altering colonic epithelial expression.

## Materials and methods

### Chemicals and dietary formulation

GLP was provided by Infinitus Co., Guangzhou, Guangdong, China, and was mainly composed of β-glucan (>90%) that contained a 1,6-linked β-D-Glcp backbone with different length branches consisting of terminal and 1,4-linked Glcp residues attached to 0–4 alternative Glc residues on the backbone (13). DSS (MW 36–50 kDa) was purchased from MP Biomedicals LLC (Santa Ana, CA, USA), and the Modified EZ Detect Fecal Occult Blood Test Kit was purchased from Qiyun Biotechnology Co., Ltd. (Guangzhou, Guangdong, China). The TIANamp Stool DNA Kit was from Tiangen Biotech Co. Ltd. (Beijing, China); the AxyPrep DNA Gel Extraction Kit was from Axygen Biosciences (Union City, CA, USA); TRIzol® reagent was from Invitrogen (Shanghai, China); the TruSeq TM RNA Sample Prep Kit was from Illumina (San Diego, CA, USA); QuantiFluor™-ST was from Promega (Beijing, China); Phusion DNA polymerase was from NEB (Beijing, China) and the short chain fatty acids standard was from Sigma (Guangzhou, Guangdong, China).

All feeds used in this study were purchased from Guangdong Medical Laboratory Animal Center (Foshan, Guangdong, China). The feeds were sterilized by Co^60^ (25 kGy) radiation. Formulation No. D12450-B was used as a basal diet, and the GLP diet was prepared by replacing 10% of corn starch in the basal diet formulation with equal amount of GLP (Table 1).

**Table 1:** Formulation of feed in two different diets (g/kg feed weight)

Raw material	Basal diet (D12045-B)	*Ganoderma lucidum* polysaccharide (GLP) diet
Casein	2,500	2,500
L-cystine	37.5	37.5
Corn starch	3937.5	3543.75
GLP	0	393.75
Maltodextrin	437.5	437.5
Sucrose	4,375	4,375
Cellulose	625	625
Soybean oil	312.5	312.5
Lard	250	250
Mineral	437.5	437.5
Vitamin	125	125
Choline	31.25	31.25

Note: Basal diet, diet of rats in Group Con; GLP diet, diet of rats in Group GLP. Group Con rats were fed a basal diet (D12450-B) with dextran sodium sulfate (DSS)-induced colitis; Group GLP rats were fed a GLP diet with DSS-induced colitis.

### Animals feeding and sample preparation

For laboratory studies, application of DSS at 40–50 kDa can induce severe murine colitis, which most closely resembles human UC (14, 15). The DSS solution was applied to establish the colitis model in this study. Twelve male Wistar rats (Specific pathogen free grade, 200–220 g, Guangdong Medical Laboratory Animal Center, Guangzhou, Guangdong, China) were housed in polypropylene cages at the Institute of Laboratory Animal Science at Jinan University, with a constant temperature (22 ± 2°C) under a 12-h:12-h light/dark cycle. During an acclimation period of 10 days, the mice were fed a basal diet (No. D12450-B) and sterilized distilled drinking water (dH_2_O). After acclimation, they were randomly divided into two groups – Group Con and Group GLP (six rats in each group) – and supplied a basal and a GLP diet, respectively, for 3 weeks. Then, the sterilized distilled drinking water was replaced by DSS solution (2.5%, w/v) for 8 days (16). During the 8-day trial, the rats in Group Con and Group GLP were continually fed the basal and the GLP diets, respectively.

The animals were euthanized by cervical dislocation after the trial. The small intestinal samples, cecal samples, and colonic epithelial cells (CECs) were immediately collected after dissection as described (17).

The animal experiments in this study were approved by the Institutional Animal Care and Use Committee of Jinan University (No. 20161223-46), and all Institutional Animal Care and Use Committee of Jinan University guidelines for the care and use of animals were followed.

### Determination of the disease activity index

The disease activity index (DAI) was measured during the trial according to a previous study (18) and was calculated based on the following equation:

DAI = (the average score of body weight decreasing rate + the average score of fecal property + the average score of hematochezia status)/3

The body weight of each group was measured by an electronic balance (*n* = 6). The fecal property was classified into normal, semiloose, and loose. Normal stools referred to a granular stool, semiloose stools referred to a paste-shaped loose stool that did not adhere to the anus or a semi-formed stool, and loose stools referred to a watery stool that adhered to the anus. The hematochezia status was classified as normal feces, feces with occult blood, and bloody feces. The normal feces, feces with occult blood (+), and feces with occult blood (++) referred to stools without visible blood that showed 3 negative, 1–2 positive, and 3 positive testing results, respectively, with the Modified EZ Detect Fecal Occult Blood Test Kit. Bloody feces referred to stool with visible blood presenting red or dark red in color (Table 2).

**Table 2:** The detailed criteria for the disease activity index scoring

Score	Body weight decrease rate	Fecal property	Hematochezia status
0	0%	Normal	Normal
1	1–5%	Semiloose (+)	Feces with occult blood (+)
2	6–10%	Semiloose (++)	Feces with occult blood (++)
3	11–15%	Loose (+)	Bloody feces (+)
4	>15%	Loose (++)	Bloody feces (++)

Note: Normal stools refer to granular stools; semiloose stools refer to paste-shaped loose stools that do not adhere to the anus or semi-formed stools; lose stools refer to watery stools that adhere to the anus. The normal feces, feces with occult blood (+), and feces with occult blood (++) referred to stool without visible blood and showed 3 negative, 1–2 positive, and 3 positive testing results with the Modified EZ Detect Fecal Occult Blood Test Kit, respectively. Bloody feces referred to stool with visible blood presenting red or dark red in color.

The Modified EZ Detect Fecal Occult Blood Test Kit was used according to the protocol described by the manufacturer. In brief, 2 g of feces was placed into 50 mL distilled water, and the test paper was then placed on the surface of the water and allowed to float for 2 min. A positive result was obtained when the color of the test paper turned cyan, and a negative result was obtained when the color remained unchanged. The tests were run three times for each sample.

### Determination of short-chain fatty acids in cecal samples

The concentrations of SCFAs in cecal samples were measured as per the method described by our previous report, using gas chromatography with a hydrogen flame detector (19). Standard curves were obtained using different concentrations of acetic, propionic, and butyric acids. The detecting conditions were as follows: the carrier gas was N_2_; the split ratio was 10:1; the flow rate was 1.5 mL/min constant; the injector temperature was 220°C; the detector temperature was 250°C; the chromatographic column was DB-WAX, and the sample volume was 1 μL.

### The 16S rDNA sequencing of gut microbiota

Small intestinal and cecal bacterial DNA from six rats in each group was extracted and analyzed in accordance with our previous report (20). Briefly, the V3–V4 region of bacterial 16S rRNA gene was amplified by PCR (polymerase chain reaction) (ABI GeneAmp 9700 model). The PCR products were examined by 2% agarose electrophoresis and then purified and quantified. The sequencing of purified amplicons was achieved through an Illumina MiSeq platform according to the standard protocols. The metagenomic analysis with MiSeq techniques and 16S rDNA sequencing of gut microbiota were analyzed according to our previous reports using QIIME software (Quantitative Insights into Microbial Ecology, v1.8.0) (21).

### RNA extraction, library preparation, and sequencing

Total RNA was extracted from the CECs using TRIzol® Reagent according to the manufacturer’s instructions after removal of genomic DNA by DNase I (Takara, Dalian, China). The quality was then determined by a 2100 Bioanalyzer (Agilent Technologies, Santa Clara, CA, USA) and quantified by ND-2000 (Thermo Fisher, Wilmington, DE, USA). Only high-quality RNA samples (concentration > 50 ng/μL, OD260/280 = 1.8~2.2, OD260/230 = 1.8~2.2, RIN > 6.5, 28S:18S ≥ 0.5) were used to construct the sequencing library. The libraries were prepared with an Illumina TruSeq TM RNA Sample Prep Kit using 5 μg of the total RNA. In brief, mRNA was purified and followed by cDNA synthesis with random hexamers (22). Then, the synthesized cDNA was subjected to end-repair. Libraries for cDNA target fragments of 200–300 bp were selected using 2% low-range ultra-agarose followed by PCR amplification using Phusion DNA polymerase for 15 PCR cycles. After quantification by TBS380, a paired-end RNA-seq sequencing library was sequenced with the Illumina HiSeq 4000 (2 × 150 bp read length).

### Read mapping

The raw paired-end reads were trimmed and quality controlled by SeqPrep and Sickle with default parameters. Then, clean reads were separately aligned to the reference genome (Rattus_norvegicus.Rnor_6.0.dna.toplevel.fa) with orientation mode using Bowtie2 software (23), with the mapping criterion as follows: sequencing reads were uniquely matched to the genome allowing up to two mismatches without insertions or deletions. Then, the region of the gene was expanded following the depths of the sites. In addition, the whole genome was split into multiple 15 kbp windows that shared 5 kbp. Newly transcribed regions were defined as more than two consecutive windows without overlapping regions of genes, where at least two reads were mapped per window in the same orientation. The sequencing data were annotated using databases of Ensembl, GO, KEGG, eggNOG, Symbol, and UniProtKB.

### Differential expression analysis and functional enrichment

To identify differentially expressed genes (DEGs) between two different samples, the expression level of each transcript was calculated according to the fragments per kilobase of exon per million mapped reads (FRKM) method. RSEM (a software package that quantifies gene and isoform abundances from single-end or paired-end RNA-Seq data) was used to quantify gene abundances (24). The R statistical package software Empirical Analysis of Digital Gene Expression in R (EdgeR) was used for differential expression analysis (25). In addition, functional enrichment analysis including KEGG was performed to identify which DEGs were significantly enriched in certain metabolic pathways at the Bonferroni-corrected *P*-value [also named false discovery rate (FDR)] ≤0.05 compared with the whole-transcriptome background by Goatools and KOBAS (a web server for gene/protein functional annotation and functional gene set enrichment) analysis (26).

### Statistical analysis

The statistical analysis of the bioinformatics data was performed by R software using the default setting. The statistical analysis for other data was performed using SPSS 20.0 software (SPSS Inc., Chicago, IL, USA). The normality of distribution was examined using the Kolmogorov–Smirnov test. The distribution was normal if the *P-*value was greater than 0.05. A two-tailed Student’s T-test (normal distribution) or two-tailed Mann–Whitney U test (abnormal distribution) was conducted to analyze two independent samples (Group Con and Group GLP). Statistical significance was set at a *P*-value less than 0.05 (*P* < 0.05). All data are presented in the text as the mean ± standard deviation (SD) if the distribution was normal or as the median (minimum value, maximum value) if the distribution was abnormal.

## Results

### Body weight, DAI scores, and cecal SCFAs

**Fig. 1:**
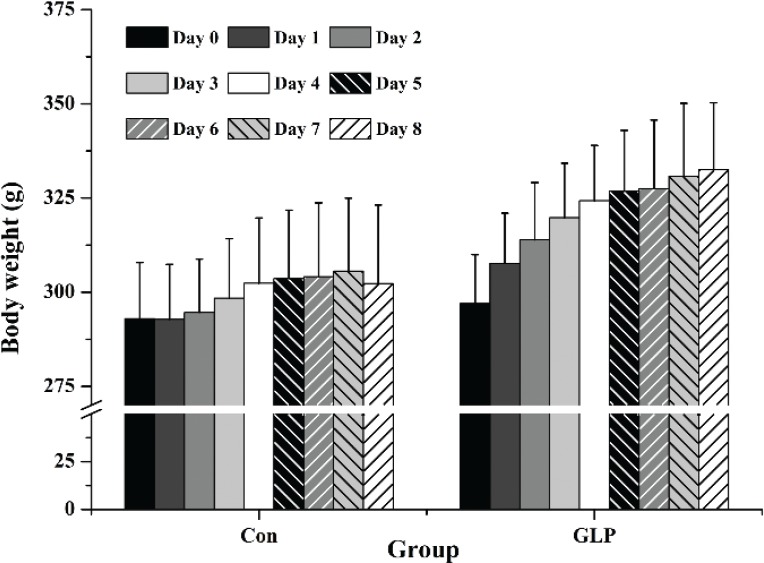
Body weight (g) of Groups Con and GLP before and during the 8-day trial. Con: Group Con rats were fed a basal diet (D12450-B) with DSS-induced colitis; GLP: Group GLP rats were fed a GLP diet with DSS-induced colitis.

The body weights of rats in Group Con and Group GLP after replacement of 2.5% DSS solution of drinking water are presented in Fig. 1. In addition, the rats in different groups showed symptoms of enteritis at different degrees, resulting in different DAI scores (Table 3). The rats did not show any symptoms on days 1–3, but they had diarrhea and loose feces on day 4, especially for those in Group Con. On day 6, feces with occult blood were found in rats from both Groups Con and GLP (three in Group Con and one in Group GLP). Bloody feces were found in two rats in Group Con on day 7 and in all rats on day 8. No rats in Group GLP were found to have hematochezia. Moreover, the body weight decreased at a rate of 1–5% in four rats in Group Con, while no body weight loss was detected in rats of Group GLP on day 8. As a result, the DAI score of Group GLP was significantly lower than that of Group Con (Table 3).

**Table 3:** The detailed disease activity index (DAI) score and description on days 4–8 during colitis induced with 2.5% dextran sodium sulfate solution

Time	Group	S1	S2	S3	DAI	Description
Day 4	Con	0	5	0	0.28 ± 0.19	2×semiloose (++), 1×semiloose (+)
	*Ganoderma lucidum* polysaccharide (GLP)	0	1	0	0.06 ± 0.14	1×semiloose (+)
Day 5	Con	0	9	0	0.50 ± 0.17	3×semiloose (++), 1×loose (+)
	GLP	0	6	0	0.33 ± 0.33	1×semiloose (++), 1×semiloose (+), 1×loose (+)
Day 6	Con	0	14	3	0.94 ± 0.36	2×loose (+) + fecal occult blood (+),1×loose (+), 1×semiloose (++) + fecal occult blood (+), 1×semiloose (++), 1×semiloose (+)
	GLP	0	14	1	0.83 ± 0.28	1×loose (+) + fecal occult blood (+), 1×loose (+), 4×semiloose (++)
Day 7	Con	0	20	11	1.72 ± 0.49	2×loose (++) + hematochezia (+), 1×loose (+) + fecal occult blood (++), 3×loose (+) + fecal occult blood (+)
	GLP	0	18	5	1.28 ± 0.25	1×loose (+) + fecal occult blood (++), 3×loose (+) + fecal occult blood (+), 2×loose (+)
Day 8	Con	4	24	24	2.89 ± 0.17	4×loose (++) + hematochezia (++) + 1–5% body weight decreasing, 2×loose (++) + hematochezia (++)
	GLP	0	19	5	1.33 ± 0.30*	1×loose (+) + fecal occult blood (++), 1×loose (++) + fecal occult blood (+), 2×loose (+) + fecal occult blood (+), 2×loose (+)

Note: DAI was calculated by using the equation *, significant difference was found when compared with Group Con on the same day (*P* < 0.05). S1, the total score of the body weight decrease rate; S2, the total score of the fecal property; S3, the total score of hematochezia status. The detailed criteria for the scoring are referred to in Table 2.

**Fig. 2:**
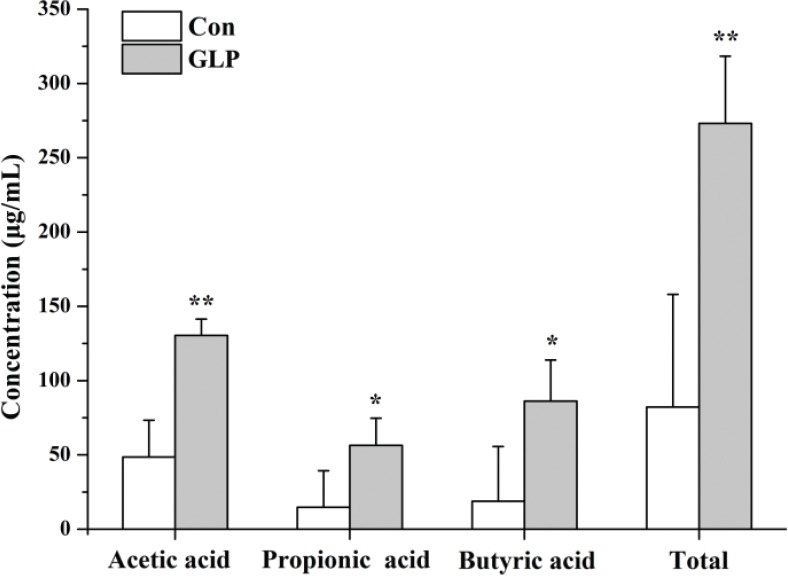
Short chain fatty acid levels in cecal contents (μg/mL) of Groups Con and GLP. Con: Group Con rats were fed a basal diet (D12450-B) with DSS-induced colitis; GLP: Group GLP rats were fed a GLP diet with DSS-induced colitis; Total acid: Sum of acetic acid, propionic acid, and butyric acid levels.

The acetic acid content in cecal contents of Group GLP was 130.41 ± 11.01 μg/mL, which was significantly higher than that of Group Con (48.54 ± 24.78 μg/mL, *P* < 0.01). Both the propionic acid and butyric acid contents were significantly higher than those in Group Con (56.41 ± 18.30 vs. 14.79 ± 24.57 μg/mL and 86.26 ± 27.62 vs. 18.73 ± 36.83 μg/mL, respectively, *P* < 0.05). As a result, the total acid content of Group GLP was much higher than that of Group Con (*P* < 0.01) (Fig. 2).

### Overall distribution and alpha-diversity of gut microbiota

**Fig. 3:**
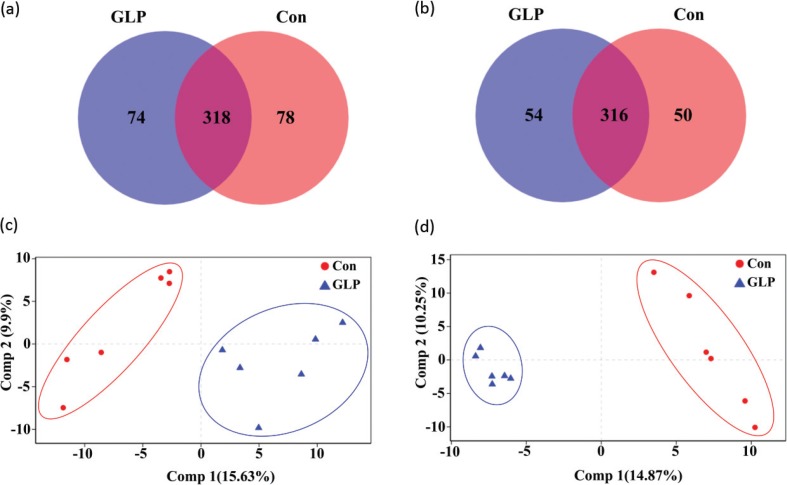
Venn diagrams and PLS-DA plots of small intestinal and cecal microbiota at the operational taxonomic unit level in rats in two different groups. (a) Venn diagram of small intestinal microbiota, (b) Venn diagram of cecal microbiota, (c) PLS-DA plots of small intestinal microbiota, and (d) PLS-DA plots of cecal microbiota. Con: Group Con rats were fed a basal diet (D12450-B) with DSS-induced colitis; GLP: Group GLP rats were fed a GLP diet with DSS-induced colitis.

In total, 225,270 sequences from small intestinal microbiota were detected in Group Con, while 233,235 sequences were found in Group GLP (Table 4). The coverage of each of these two groups was 1.00 ± 0.00, indicating that the sequencing depth was sufficient (Table 4). The α-indices at the operational taxonomic unit (OTU) level, including Shannon, Simpson, Abundance-based Coverage Estimators (ACE), and Chao, were similar between both groups (*P* > 0.05) (Table 3). Group GLP had 74 unique OTUs and shared 318 OTUs with Group Con (Fig. 3a). Based on the Partial Least Squares-Discriminant Analysis (PLS-DA) plots, sample dots of Group Con were located on the left, while those of Group GLP were located on the right, with a clear boundary between them (Fig. 3c).

**Table 4:** Total number of sequences and alpha-diversity indices (at operational taxonomic unit level) of small intestinal and cecal microbiota

Group	Seq_Num	Coverage	Shannon	Simpson	ACE	Chao
Con^a^	225,270	1.00 ± 0.00	2.16 ± 0.97	0.31 ± 0.24	272.71 ± 67.83	259.09 ± 71.33
*Ganoderma lucidum* polysaccharide (GLP)^a^	233,235	1.00 ± 0.00	2.17 ± 0.69	0.27 ± 0.16	252.31 ± 58.13	238.27 ± 68.31
Con^b^	211,359	1.00 ± 0.00	3.11 ± 0.54	0.13 ± 0.10	261.59 ± 30.34	290.51 ± 66.00
GLP^b^	221,868	1.00 ± 0.00	3.23 ± 0.19	0.09 ± 0.02	269.07 ± 40.21	268.03 ± 42.02

Note: Con: Group Con rats were fed a basal diet (D12450-B) with dextran sodium sulfate (DSS)-induced colitis; GLP: Group GLP rats were fed a GLP diet with DSS-induced colitis. Seq_num refers to the total number of sequences in a specific group.

a Referred to the small intestinal microbiota;

b referred to the cecal microbiota.

There were 211,359 and 221,868 sequences of cecal microbiota found in Groups Con and GLP, respectively (Table 4). The coverage of these two groups also indicated that the sequencing depth was sufficient. Similarly, no significant difference was found in the four α-indices at the OTU level (Table 4). Both groups shared 316 OTUs, and 50 and 54 unique OTUs were found in Groups Con and GLP, respectively (Fig. 3b). In the PLS-DA plots of cecal microbiota, a clearer boundary between Groups Con and GLP was obtained compared with the PLS-DA plots of the small intestinal microbiota. Sample dots of Group Con were located on the right, while those of Group GLP were located on the left (Fig. 3d).

### Composition of small intestinal and cecal microbiota at the phylum and genus levels

**Fig. 4:**
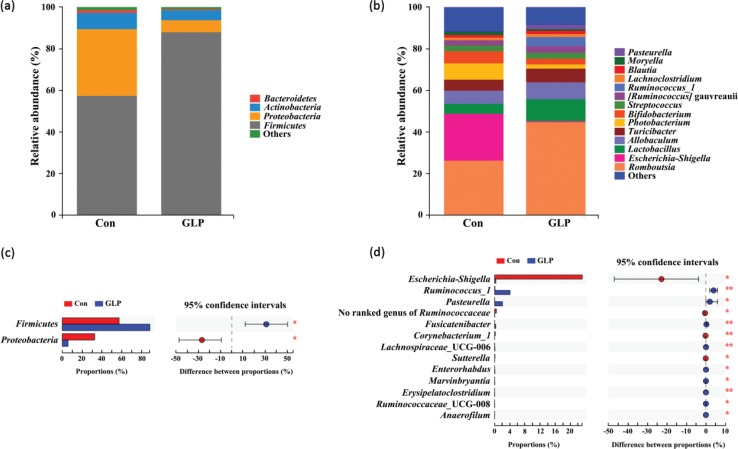
The compositions of small intestinal microbiota at the phylum and genus levels. (a) A bar chart of the small intestinal microbiota at the phylum level, (b) a bar chart of the small intestinal microbiota at the genus level, (c) comparison of small intestinal microbiota at the phylum level, and (d) comparison of small intestinal microbiota at the genus level. * and **: Significant or highly significant differences were detected (*P* < 0.05 or *P* < 0.01). Con: Group Con rats were fed a basal diet (D12450-B) with DSS-induced colitis; GLP: Group GLP rats were fed a GLP diet with DSS-induced colitis.

**Fig. 5:**
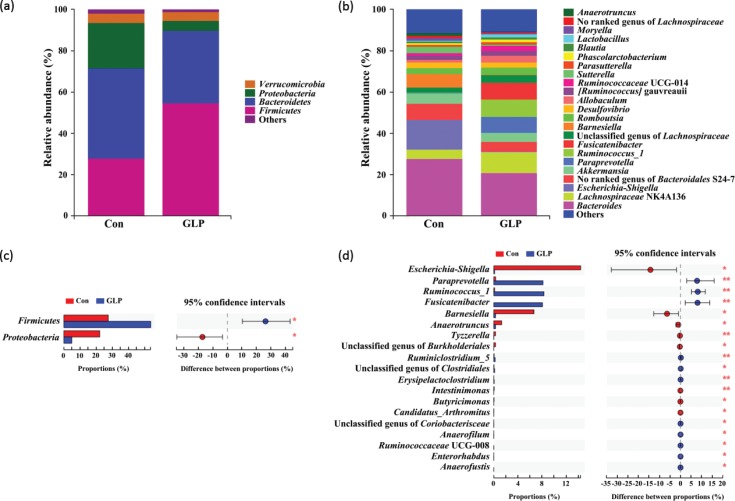
The compositions of cecal microbiota at the phylum and genus level. (a) Bar chart of cecal microbiota at the phylum level, (b) bar chart of cecal microbiota at the genus level, (c) comparison of cecal microbiota at the phylum level, and (d) comparison of cecal microbiota at the genus level. * and **: Significant or highly significant differences were detected (*P* < 0.05 or *P* < 0.01). Con: Group Con rats were fed a basal diet (D12450-B) with DSS-induced colitis; GLP: Group GLP rats were fed a GLP diet with DSS-induced colitis.

The relative abundance (RA) of small intestinal and cecal microbiota of Groups Con and GLP at both the phylum and genus levels is presented here; RA less than 1% was classified as other (Figs. 4a, b and 5a, b).

In the small intestine, the RA of *Proteobacteria* in Group Con was significantly higher than that in Group GLP, while the RA of *Firmicutes* in Group GLP was significantly higher than that in Group Con at the phylum level (*P* < 0.05) (Fig. 4c). At the genus level, the RA of *Escherichia-Shigella* (23.13 ± 29.77%), no ranked genus of *Ruminococcaceae* (0.47 ± 0.71%), *Corynebacterium_1* (0.18 ± 0.17%), and *Sutterella* (<0.1%) in Group Con was significantly higher than those in Group GLP (*P* < 0.01 or *P* < 0.05) (Fig. 4d). In addition, the RA of *Ruminococcus_1* (4.07 ± 2.67%), *Pasteurella* (2.05 ± 4.82%), *Fusicatenibacter* (0.29 ± 0.24%), *Lachnospiraceae*_UCG-006 (<0.1%), *Enterorhabdus* (<0.1%), *Marvinbryantia* (<0.1%), *Erysipelatoclostridium* (<0.1%), *Ruminococcaceae*_UCG-008 (<0.1%), and *Anaerofilum* (<0.1%) in Group GLP was significantly higher when compared with those in Group Con (*P* < 0.01 or *P* < 0.05) (Fig. 4d).

Similarly, in the cecum, Group Con had a significantly higher RA of *Proteobacteria*, while Group GLP had a significantly higher RA of *Firmicutes* at the phylum level (*P* < 0.05) (Fig. 5c). Regarding the genus level, the RA of *Escherichia-Shigella* (14.41 ± 21.63%), *Barnesiella* (6.67 ± 8.37%), *Anaerotruncus* (1.32 ± 1.31%), *Tyzzerella* (0.29 ± 0.14%), unclassified genus of *Burkholderiales* (0.30 ± 0.30%), *Intestinimonas* (<0.1%), *Butyricimonas* (<0.1%), *Candidatus* Arthromitus (<0.1%) in Group Con was significantly higher than those in Group GLP (*P* < 0.01 or *P* < 0.05) (Fig. 5d). Moreover, the RA of *Paraprevotella* (8.16 ± 9.76%), *Ruminococcus_1* (8.32 ± 4.24%), *Fusicatenibacter* (8.08 ± 7.76%), *Ruminiclostridium_5* (0.23 ± 0.14%), unclassified genus of *Clostridiales* (0.15 ± 0.09%), *Erysipelatoclostridium* (<0.1%), unclassified genus of *Coriobacteriaceae* (<0.1%), *Anerofilum* (<0.1%), *Ruminococcaceae* UCG-008 (<0.1%), *Enterorhabdus* (<0.1%), and *Anaerofustis* (<0.1%) in Group GLP was significantly higher than those in Group Con (*P* < 0.01 or *P* < 0.05) (Fig. 5d).

### The gene expression profile of CECs

**Fig. 6:**
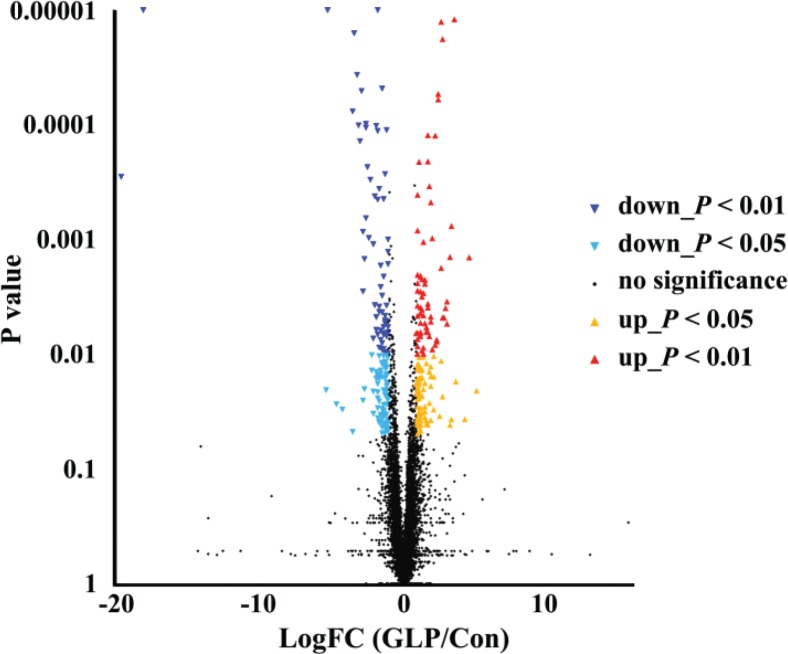
Volcano charts of gene regulations by comparison between Group GLP and Group Con.

In total, 299 genes were differentially expressed, including 150 that were upregulated and 149 that were downregulated (FDR < 0.05) (Fig. 6). The detailed information of each DEG is listed in Supplementary Table 1. These 299 genes were enriched in 187 KEGG pathways (Supplementary Table 2). Among them, six of these pathways were screened, including three related to signal transduction, two related to the immune system, and one related to immune diseases (Table 5). Eleven DEGs belonging to these six KEGG pathways were screened, which included six upregulated and five downregulated genes (Table 6).

**Table 5:** Information on KEGG pathways related to inflammation

Term	ID	Level 1	Level 2
NF-κB signaling pathway	Ko04064	Environmental information processing (EIP)	Signal transduction
Tumor Necrosis Factor (TNF) signaling pathway	Ko04668	EIP	Signal transduction
Toll-like receptor signaling pathway	Ko04620	Organismal system (OS)	Immune system
Janus Kinase/Signal Transducers and Activators of Transcription (JAK/STAT) signaling pathway	Ko04630	EIP	Signal transduction
T cell receptor signaling pathway	Ko04660	OS	Immune system
Inflammatory bowel disease	Ko05321	Human diseases	Immune diseases

**Table 6:** Information on regulated differentially expressed genes enriched in inflammation-related KEGG pathways

Gene	Regulation	LogFC(Glu/Con)	KEGG pathway	NR_description
*Ccl5*	Up	2.83	Ko04668; Ko04620	C-C motif chemokine 5 precursor
*Cd3e*	Up	1.25	Ko04660	PREDICTED: T cell surface glycoprotein CD3 epsilon chain isoform X1
*Cd8a*	Up	1.25	Ko04660	T cell surface glycoprotein CD8 alpha chain precursor
*Il21r*	Up	1.18	Ko04630; Ko05321	Interleukin-21 receptor precursor
*Lck*	Up	1.03	Ko04064; Ko04660	PREDICTED: proto-oncogene tyrosine-protein kinase LCK isoform X2
*Trbv*	Up	1.26	Ko04064; Ko04660; Ko05321	Tcrb protein
*Ccl3*	Down	−1.80	Ko04620	C-C motif chemokine 3, partial
*Gro* (*Cxcl1, 2, 3*)	Down	−2.38	Ko04668	Chemokine (C-X-C motif) ligand 1
*Il11*	Down	−2.54	Ko04630	Interleukin-11 precursor
*Mhc2*	Down	−1.86	Ko05321	rCG60985, isoform CRA_b
*Ptgs2* (*Cox2*)	Down	−1.56	Ko04064; Ko04668	Prostaglandin G/H synthase 2 precursor

Note: NR_description, the description of genes based on the NR database of NCBI.

## Discussion

The consumption of prebiotics, particularly dietary fibers, has been confirmed to be associated with many health benefits to the host, such as lowering the rate of cardiovascular disease, assisting with weight loss, and maintaining gut health (27). It is believed that the consumption of dietary fiber stimulates the growth of probiotic bacteria and produces more SCFAs (28), which have distinct physiological effects, including shaping of gut environment, acting as energy sources for host cells and intestinal microbiota, and participating in host-signaling mechanisms (29). As found in our manuscript, relatively high amounts of SCFAs, including acetic acid, propionic acid, and butyric acid, were detected in rats fed with the GLP diet. Propionate and butyrate can be utilized by hosts (29) to improve intestinal health, such as retarding the progression of colon inflammation and cancer development by complex regulation mechanisms, including the modulation of cytokines within some inflammation-related pathways (30). Considering the significantly higher content of SCFAs and the significantly lower DAI score in this study, the consumption of GLP should also be beneficial to rat colon health.

As mentioned above, dietary fibers and gut microbiota were closely related. Some glucans, including β-glucan, can promote the growth of *Ruminococcus* as it is capable of decomposing dietary polysaccharides (31, 32). Therefore, the higher amount of *Ruminococcus_1* in both the small intestine and cecum may have been responsible for the higher production of SCFAs in this experiment. Moreover, *Paraprevotella* also possessed saccharolytic capacity (33), making it possible to hydrolyze GLP to produce SCFA to improve colon health. Other important profiles of gut microbiota in the GLP-fed rats also indicated the benefits of GLP. For example, one specific strain of *Fusicatenibacter* induced the production of anti-inflammatory cytokines (34). Thus, the higher amount of *Fusicatenibacter* in the present research may promote the potential for GLP to improve colon health. Furthermore, GLP also reduced the amount of some pathogens, such as *Escherichia–Shigella,* both in the small intestine and cecum, which had been shown to be closely related to acute diarrhea (35) and maintained high amounts in the colon of IBD patients (36).

Based on previous results of transcriptional analysis, UC has been found to alter the expression of intestinal epithelial cells (37), especially in the genes involved in immune response (38). In addition, the alteration of specific bacteria in gut, together with the high contents of SCFAs, affected the gene expression of CECs. As a result, six KEGG pathways that were considered to be inflammation or immune related were selected (39–43), and 11 genes, including six upregulated and five downregulated genes, were further screened. Among the six upregulated genes, *Lck* was considered to activate and mature T lymphocytes (44). *Cd8a* was also crucial for the production of T lymphocytes, while *Cd3e* was the essential component of the T cell receptor (TCR) complex for antigens (45, 46). Therefore, these four genes may improve the immunity of the colon in rats. Moreover, upregulated *Ccl5* was an important link between platelet activation and neutrophil recruitment in acute colitis, and the immunoneutralization of *Ccl5* reduced tissue damage (47). However, *Trbv* and *Il21r* were upregulated, but their roles in the colitis rats fed with the GLP diet remained unclear. Further research should be performed to clarify their functions.

Furthermore, five genes were downregulated by GLP. *Ccl3* is commonly highly expressed in inflammation, and *Il-11* is elevated in IBD patients; hence, its downregulation may become a therapeutic target for IBD treatment (48, 49). The expression of genes in the *Gro* family and *Ptgs* (*Cox2*) was increased in colon cancer subjects, displaying an association between these genes and cancer risk (50, 51). It thus can be inferred that GLP may reduce the risk of colon cancer under the circumstances of colitis. Literature mentioning the relationship between *Mhc2* and colitis is scarce, but as one of the genes included in the KEGG pathways for IBD, its downregulation by GLP may also possibly alleviate colitis. Taken together, GLP could enhance immunity and reduce the inflammatory response and colon cancer risk when compared with the basal diet, as indicated by the gene expression profile.

However, the results from the omics analysis were not validated by either quantitative polymerase chain reaction (qPCR) or Western blot analysis and thus only present an overview of the effects of GLP consumption. In addition, more research should be performed to validate the most suitable dose and function of GLP.

## Conclusion

In general, GLP consumption in colitis rats significantly lowered the DAI and produced markedly more SCFAs in the cecum than a basal diet (D12450-B). This may be mainly due to the increasment of SCFA-producing bacteria, including *Ruminococcus_1*, and the reduction of pathogens, such as *Escherichia-Shigella*, in both the small intestine and cecum. SCFAs and the altered gut microbiota further regulated the expression of 11 genes enriched in six KEGG pathways related to inflammation, resulting in enhancement of immunity and reduction of inflammatory response and colon cancer risk. Therefore, GLP could alleviate DSS-induced colitis, which most closely resembles human UC and thus may have potential for UC relief. Further studies are needed to determine the most suitable dose for humans after using GLP as a beneficial health product for people who have intestinal dysfunction or colitis.

## Supplementary Material

The detailed criteria for the DAI scoring

The Information of all differentially expressed genes (DEGs) between Group Con and Group GLP screened out in this study

The Information of all KEGG pathways in which the DEGs enriched in this study
