# Supplementary material for: Ganoderma lucidum polysaccharide improves rat DSS-induced colitis by altering cecal microbiota and gene expression of colonic epithelial cells
Source: Food Nutr Res. 2019 Feb 12;63:10.29219/fnr.v63.1559. doi: 10.29219/fnr.v63.1559 (PMC6387425; doi:10.29219/fnr.v63.1559)
Supplement: The detailed criteria for the DAI scoring [file FNR-63-1559-s001.docx]

Table S1 The detailed criteria for the DAI scoring

| Score | Body weight decreasing rate | Fecal property | Hematochezia status |
| --- | --- | --- | --- |
| 0 | 0% | normal | normal |
| 1 | 1-5% | semi-loose (+) | fecal occult blood (+) |
| 2 | 6-10% | semi-loose (++) | fecal occult blood (++) |
| 3 | 11-15% | loose (+) | hematochezia (+) |
| 4 | >15% | loose (++) | hematochezia (++) |

Note: The normal stools refers to the granular stool; Semi-loose stools refers to the paste shaped loose stool which does not adhere to the anus, or the semi formed stool; Loose stools refers to the watery stool adhered to the anus.
